# Center of mass in complex networks

**DOI:** 10.1038/srep40982

**Published:** 2017-01-20

**Authors:** Chuanji Fu, Yachun Gao, Shimin Cai, Hongchun Yang, Chun Yang

**Affiliations:** 1 School of Physical Electronics, University of Electronic Science and Technology of China, Cheng Du 610054, P.R. China; 2 Web Sciences Center & Big Data Research Center, University of Electronic Science and Technology of China, Cheng Du 610054, P.R. China; 3 School of Mathematical Science, University of Electronic Science and Technology of China, Cheng Du 610054, P.R. China

## Abstract

Network dynamics is always a big challenge in nonlinear dynamics. Although great advancements have been made in various types of complex systems, an universal theoretical framework is required. In this paper, we introduce the concept of center of ‘mass’ of complex networks, where ‘mass’ stands for node importance or centrality in contrast to that of particle systems, and further prove that the phase transition and evolutionary state of the system can be characterized by the activity of center of ‘mass’. The steady states of several complex networks (gene regulatory networks and epidemic spreading systems) are then studied by analytically calculating the decoupled equation of the dynamic activity of center of ‘mass’, which is derived from the dynamic equation of the complex networks. The limitations of this method are also pointed out, such as the dynamical problems that related with the relative activities among components, and those systems that consist of oscillatory or chaotic motions.

There is growing fascination with behaviors of the high-dimensional complex system^1^,^2^,^3^,^4^,^5^,^6^, particularly the phase transitions in these systems with huge components interacting, which usually described by the coupled dynamical units on the complex network, for example, epidemic spreading^7^, neuronal networks^8^, the Kuramoto model^9^, systems of self-driven particles^10^, percolation or cascading procedure^11^,^12^,^13^,^14^,^15^,^16^,^17^, Ising model^18^,^19^,^20^,^21^,^22^,^23^ and ecosystems^24^,^25^,^26^ etc. These dynamical systems are usually in different states under different environmental conditions, such as the healthy and endemic states in epidemic spreading model, free flow and congestion states in transportation systems or communication networks, non-coherence and coherence states in synchronization systems, survival and extinction states in ecosystems. Therefore, a large number research effort has been devoted to understanding the dynamic behaviors of these systems, and obtaining the ways to foresee the universal existence critical transitions phenomenon^27^,^28^,^29^,^30^,^31^,^32^,^33^,^34^,^35^,^36^,^37^.

One significant issue is the interplay between phase transition and network topology, and current theoretical achievements are mainly obtained based on mean-field theory and renormalization group theory^3^,^4^,^5^,^7^,^27^. However, these theories are still too complicated to give accurate approximation in heterogeneous coupled systems, in which the interacting complex networks are usually highly heterogenous, i.e., degree distribution is very inhomogeneous among the components(nodes), although a few achievements are obtained to deal with these heterogeneous structure^28^.

Recently, Gao *et al*.^1^ proposed an elaborate method, coding the microcosmic interactions information between components into a single parameter, therefore the multi-dimensional coupled nonlinear equations are simply reduced into an 1D equation, which could be easily used to predict the universal phase transitions of the complex system. This method provides an analytical framework to study dynamic behaviors and phase transitions of the complex system. Nevertheless, although the validity is verified by amount of experiments, it still remains to be given a clear physical interpretation. Moreover, in order to promote this method to generalized dynamical processes on networks other than resilience, it should be to figure out the applicability and limitation of the reduced 1D dynamical equation.

Here we give a clear physical interpretation by introducing the concept of center of ‘mass’ in complex systems. And it is easy to find that the reduced 1D dynamical equation is actually the equation of the motion of the center of ‘mass’. This method captures the whole translational behavior of the dynamical system, which is the origin of universality. Therefore, it is no longer applicable to the dynamical problems that related with the relative activities among components such as coherence and synchronization of complex systems. Also, it is valid only in the case that the interaction functions between nodes have symmetric component.

## Results

### Center of ‘mass’ in complex network

For a system with *N* components(nodes), the dynamic process can be expressed by differential equation of activity *x*_*i*_ of node *i* as


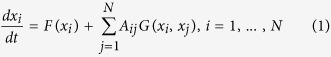


where *A*_*ij*_ denotes the weighted adjacent matrix, representing the direction and strength of the interaction. Accordingly, 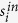 and 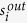, the ingoing and outgoing weighted degree of node *i*, are defined as 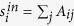 and 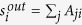, respectively. *G*(·,·) is the pairwise interaction, which is determined by fundamental interaction rule of the specified system and usually has the same form for any pairs of nodes as in this paper. The first term *F(x*_*i*_) on the right-side hand represents self-dynamics of node *i* without influences from other nodes, while the second term comes from interactions between *i* and its neighbours.

We treat the dynamical system on the network as a system of particles, where the nodes and edges of complex networks are regarded as particles and interactions of the system, respectively. In heterogenous complex networks, centrality and properties of nodes usually varies from each other since permutation symmetry breaks, we therefore could introduce a parameter to characterize this distinctiveness of node *i*, denoted as *w*_*i*_, which reflects the difficulty of its activity *x*_*i*_ converted by other nodes, resembling the concept of mass in Newtonian mechanics as intrinsic properties of a particle. The specific definition of *w*_*i*_ is not unique, depending on the topological structural of studied networks. Generally speaking, it characterize the node’s centrality(i.e., node importance) in complex networks, such as degree centrality, betweenness centrality, eigenvector centrality, etc.

The *w*_*i*_ and activity *x*_*i*_ can be regarded as the ‘mass’ and ‘velocity’ of the *i*th node(particle), respectively. Thus, the equation of motion for the *i*th particle can be written as


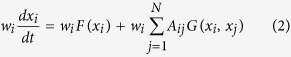


where *w*_*i*_*F(x*_*i*_) stands for the external force, and the second term is the total internal force acting on the *i*th particle due to the other particles of the system. It is noted that the internal force *f*_*ij*_ = *w*_*i*_ *A*_*ij*_ *G(x*_*i*_, *x*_*j*_) has slightly different from actual physical system, which usually does not obey Newton’s Third law, but it can be divided into anti-symmetrical and symmetrical parts as,


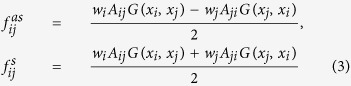


Following the research framework of Newtonian mechanics, it is convenient to study dynamics of the center of mass when describing the translational movement of the whole system. The equation of the motion for the center of mass can be obtained by summing over all particles,


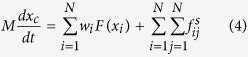


where 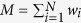 is the ‘mass’ for the center of ‘mass’ (thus also the entire ‘mass’ of the system), whose velocity is 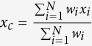. The ‘position’ of center of ‘mass’ is not concerned, but one can always get the position by integration of velocity *x*, thus the center of mass is actually in the state space. In contrast to the anti-symmetrical part of internal forces, the symmetrical part of internal forces, which does not obey Newton’s Third Law, has effect on the motion of center of mass.

The steady state of the center of ‘mass’, which captures the whole translational behavior of the dynamical system, can be changed by the coupling strength or structure precisely because of the symmetrical part of internal forces. Therefore, it can universally describe the change of the system’s state with the various environment. On the other hand, relative motion between any two particles cannot be reflected by the motion of center of ‘mass’, thus such method is not valid for the problems related with relative motion like coherence and synchronization phenomena. For example, it can not describe the phase transition between non-coherence and synchronization states of the Kuramoto model, as discussed in ref. 9.

In general, any relevant translational properties in complex systems can be described by the motion of center of ‘mass’, including oscillating and chaotic motion. However, it is very difficult to get any analytical results from equation (4), since it depend on the state of motion of all particles. To obtain certain analytical results, it can be decoupled in the following procedure. With Taylor series expansion, the two terms in the right-side hand of equation (4) can be expressed as


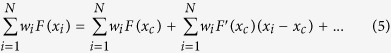



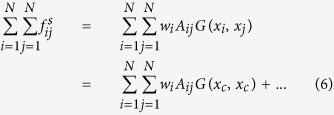


Based on the definition of *x*_*c*_, the first-order term of equation (5) 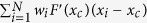 equals zero. Define 
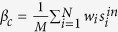
. Then the zero-order approximation of equation (4) yields,


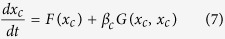


This equation is the generalized version of the equation (7) in ref. 1, where *x*_*ef f*_ is a special case of *x*_*c*_ with *w*_*i*_ being chosen as the outgoing weighted degree 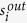, the most commonly used characterization of node importance. We show here this equation has an accuracy of zero-order approximation, but it has merits of brief description by mapping multi-dimensional complex system into one-dimension description.

### Center of ‘mass’ in gene regulatory systems

Directly from this equation, one can describe the whole motion of the global system, i.e., the common evolutionary component of all nodes, by the state of center of ‘mass’. However, this method is an zero-order Taylor approximation, to test and verify the above deduction and idea, we take the same example studied in ref. 1, of the dynamics of gene regulatory networks which evolves following the Michaelis-Menten equation


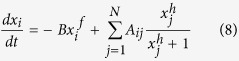


**Figure 1:**
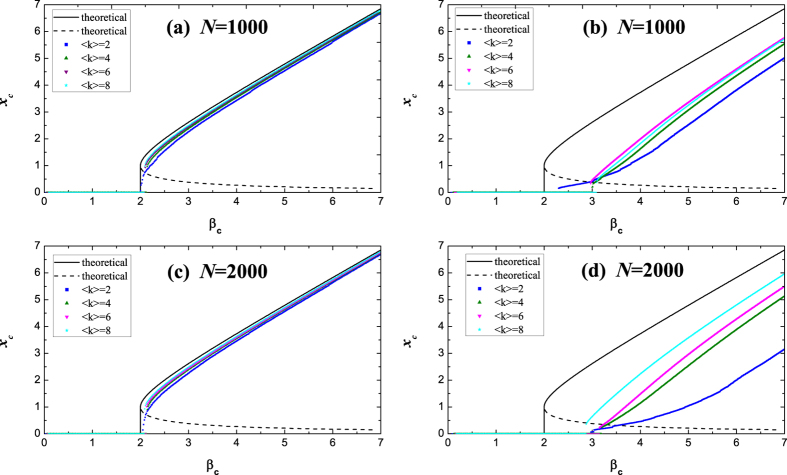
Phase transition in gene regulatory networks, with **(a)** ER random networks with size of *N* = 1000, (**b**) BA scale-free networks with size of *N* = 1000, (**c**) ER random networks with size of *N* = 2000 and (**d**) BA scale-free networks with size of *N* = 2000. The mass *w*_*i*_ is the outgoing weighted degree 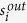 of node *i* in this figure.

Here we set *B* = 1, *f* = 1 and *h* = 2, and *A*_*ij*_ is directed and weighted, including the influence of both promoters and inhibitors. In order to investigate the influence of network topology, both Erdös-Rényi(ER)^38^ and Barabási-Albert(BA)^39^ scale-free networks with uniform weights are studied. The steady state of center of ‘mass’ *x*_*c*_ of the gene regulatory network versus various value of *β*_*c*_ can be obtained by solving equation (8) with different values of the weight, as the dots shown in Fig. 1. The results indicate that for ER random networks with various average degrees, all transition curves almost collapse into the theoretical results (see the left two graphs in Fig. 1), and the results are valid regardless of the network size from Fig. 1(a) and (c).While for BA scale-free network, the theoretical transition points are inconsistent with experimental results, and offset also exists in networks with different average degrees or different sizes(see the right two graphs in Fig. 1). The difference between theoretical and simulated results for BA network comes from the zero-order approximation in equation (7), due to the large variance of {*x*_*i*_, *i* = 1, 2, …, *N*}. Therefore, one should be very cautious with the accuracy of the theoretical result, which is highly contingent on the network topology. Most recently, Tu *et al*.^40^ give a mathematical condition when the method may be applied from error analysis.

**Figure 2:**
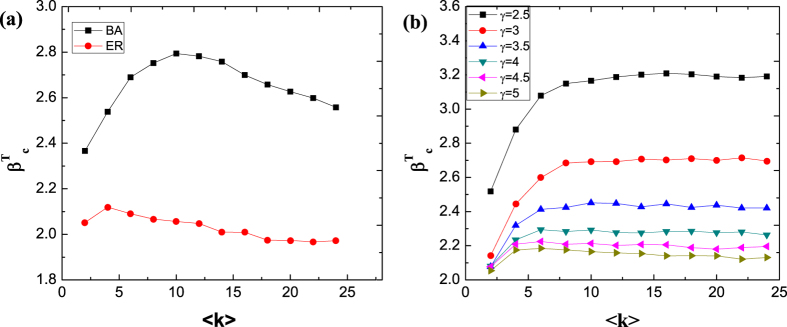
The threshold value 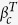 of the phase transition versus various average degrees in gene regulatory networks, with (**a**) ER random and BA scale-free networks and (**b**) scale-free networks with various degree exponent *γ*. The mass *w*_*i*_ is the outgoing weighted degree 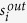 of node *i* in this figure. All the network size is *N* = 1000, and the simulations are averaged over 50 repetitions in each case.

In order to find out how average degrees 〈*k*〉 effect on the results, we study the threshold value 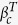 of phase transition as a function of 〈*k*〉 in three classes of networks with different structures. In Fig. 2(a), ER and BA networks are compared. It is shown that the phase transition threshold almost keeps constant in ER network, although a slightly rising at small 〈*k*〉 exists, which is in accordance with Fig. 1(a). In contrast, 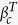 of BA scale-free network first increase significantly at 〈*k*〉 = 10, after which it decrease slowly when the average degree 〈*k*〉 increases. Moreover, the threshold value 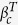 of the BA network are higher than ER network for all 〈*k*〉, because of the higher heterogeneity of the topology of BA network.

We further investigate the effect of heterogeneity on the precision of equation (7) by constructing scale-free network with tunable degree exponent *γ*. Figure 2(b) gives the threshold value 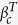 of phase transition with different power law exponent of the degree distribution versus various average degrees. As we can see, the threshold value 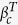 all increase monotonically to a constant value for various exponents with the increase of the average degree, and has higher value with the more heterogeneous network. It stems from the heterogeneity of the scale-free network, thus the zero-order approximation of the analytical result is insufficient for the actual system. It is also noted that the value of 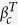 for networks with 〈*k*〉 〉 15 are almost the same, indicating that although the precision of zero-order approximation of equation (7) is not enough, *x*_*c*_ can be selected as a good order parameter. All these results indicate that the phase transition of the complex system depends on more than one parameter *β*_*c*_. And in most cases, it is even difficult to determine the dimension of the control parameter space, which makes it an challenging but attractive work.

**Figure 3:**
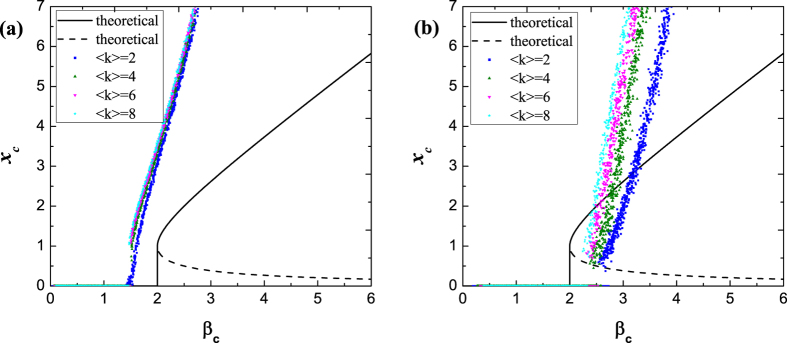
Phase transition in gene regulatory networks, with (**a**) ER random networks and (**b**) BA scale-free networks. The mass *w*_*i*_ is betweenness of node *i* in this figure, and all the network size is *N* = 1000.

It is important to highlight that the selection of *w*_*i*_ is non-exclusive, hence other centrality should also be available, and the optimal centrality could vary in different complex systems, depending on the specific dynamical problem. In Fig. 3, similar experiments are carried out for *w*_*i*_ representing betweenness centrality. It shows that universality also holds for ER random networks, but there is a deviation of predicted results from experimental results, indicating that outgoing weighted degree is more efficient in the gene regulatory networks.

### Center of ‘mass’ in epidemic spreading dynamics

In fact, the approximation equation (7) has more general application in other dynamical systems than the problem of resilience. As another example, the susceptible-infected-susceptible(SIS) model, which describe the spread of the disease among populations, can also certify the effectiveness of the this method. SIS model has been extensively studied with several theoretical approaches ranging from approximate mean-field theories to exact methods, yet as we will see, the same epidemic threshold of SIS model can be easily obtained based on the approximation method. The dynamical equation for the SIS model, under the approximation of individual-based mean-field theory, can be written as following,


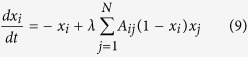


where *x*_*i*_(*t*) indicates the probability that a node is infected at time *t*, and *λ* is the effective transmission rate. Let 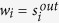, and in unweighted network 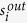 is equivalent to *k*_*i*_, the node degree, thus the equation (9) can be reduced as following,


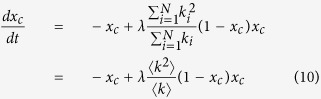


Hence a transcritical bifurcation occurs at 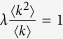, which gives the same epidemic threshold 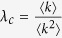 as obtained according to heterogeneous mean-field theory^41^.

Notice that recent works^42^,^43^ having improves the precision of epidemic threshold values, which is not surprising, since these results are obtained by considering the factors that is not included in zero-order approximation but in high-order terms.

### Center of ‘mass’ in chaotic systems

According to above examples, we have proved the suitability of approximate analytical expression of center of ‘mass’ in dynamic systems, however, this method should be inspected very carefully when extended to other dynamic processes because they may have particular evolutional properties. The equation (7) is a first-order autonomous dynamic system. The behaviors of the first-order autonomous system are very limited. All trajectories either monotonically approached a fixed point, or diverged to ±∞. Therefore, the approximate equation (7) can not describe those systems that consist of oscillatory or chaotic solutions.

Nevertheless, the motion of center of ‘mass’ can be arbitrary, since it can monotonically tend to the steady state, periodic, and even chaotic motion. Therefore, center of ‘mass’ can be selected as a good order parameter in these systems. As an instance, we study the chaotic Rössler system^44^


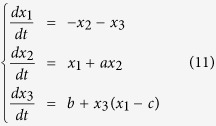


**Figure 4:**
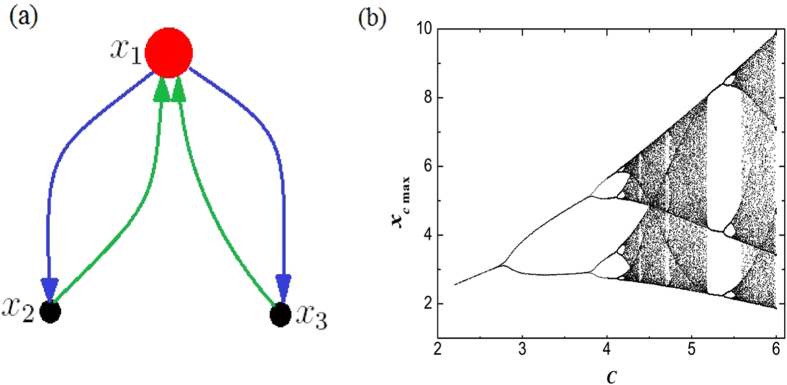
(**a**) Network corresponding to the Rössler system.(**b**) Bifurcation diagram in Rössler system for *a* = *b* = 0.2, and over a variety of *c* values. Over each *c*, the number of local maxima *x*_*c*_ on the attractor is given, reflecting the period of the attractor. The phase transitions of period-doubling to chaos (at 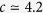) and the large period-3 window are observed.

This is a 3-Dimensional system, which could be mapped into a simple network with only 3 nodes, as seen in Fig. 4(a). We examine this model for parameter values *a* = *b* = 0.2 and a series values of *c*, in order to reveal the phase transition of orbits. Let 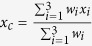 with 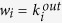, we plot all the local maxima *x*_*c*_ on the attractor above each values of *c*, where the number of different maxima *x*_*c*_ tells us the period of the attractor, as shown in Fig. 4(b). The bifurcation diagram perfectly captures the phase transition of the orbits. We can clearly see the period-doubling bifurcation route to chaos and the large period-3 window with increasing *c*, verifying the effectiveness of mass of center as an order parameter in the characterization of phase transition in chaotic systems.

## Discussion

In this paper, we introduce the concept of center of ‘mass’ for system on complex network, where ‘mass’ stands for node importance or centrality. Consequently, the dynamics and phase transition of the system can be characterized by the activity of center of ‘mass’, which describes the co-movement part of the whole complex system, instead of the dynamical problems that related with the relative activities among components such as coherence and synchronization of complex networks. We also show that only symmetrical component of the interactions has effect on the dynamics of center of mass.

By zero-order Taylor approximation, the nonlinear dynamic equation of center of mass is reduced to a 1D first-order autonomous system, from which the steady state and evolutionary dynamics of center of mass can be solved out. This equation is the generalized version to the effective 1D equation in Gao’s paper, and we give a clear physical interpretation, also further point out the limitations of this method. The result indicate that the precise is good for the homogeneous networks, but not sufficient for the heterogeneous networks. In addition, we found that in different topology of networks, there are other alternatives than degree when choosing centrality. It may shed light on establishing a bridge between classical mechanics and complex systems.

## Additional Information

**How to cite this article**: Fu, C. *et al*. Center of mass in complex networks. *Sci. Rep.* **7**, 40982; doi: 10.1038/srep40982 (2017).

**Publisher's note:** Springer Nature remains neutral with regard to jurisdictional claims in published maps and institutional affiliations.
